# Promising Results from Alzheimer's Disease Passive Immunotherapy Support the Development of a Preventive Vaccine

**DOI:** 10.34133/2019/5341375

**Published:** 2019-05-19

**Authors:** D. J. Marciani

**Affiliations:** Qantu Therapeutics, Inc., 612 E. Main Street, Lewisville, TX 75057, USA

## Abstract

The apparently near-term effects of the monoclonal antibody BAN2401 in slowing the progression of prodromal Alzheimer's disease (AD) has created cautious optimism about the therapeutic use of antibodies that neutralize cytotoxic soluble amyloid-*β* aggregates, rather than removing plaque. Plaque being protective, as it immobilizes cytotoxic amyloid-*β*, rather than AD's causative agent. The presence of natural antibodies against cytotoxic amyloid-*β* implies the existence of a protective anti-AD immunity. Hence, for vaccines to induce a similar immunoresponse that prevents and/or delays the onset of AD, they must have adjuvants that stimulate a sole anti-inflammatory Th2 immunity, plus immunogens that induce a protective immunoresponse against diverse cytotoxic amyloid-*β* conformers. Indeed, amyloid-*β* pleomorphism may explain the lack of long-term protection by monoclonal antibodies that neutralize single conformers, like aducanumab. A situation that would allow new cytotoxic conformers to escape neutralization by previously effective monoclonal antibodies. Stimulation of a vaccine's effective immunoresponse would require the concurrent delivery of immunogen to dendritic cells and their priming, to induce a polarized Th2 immunity. An immunoresponse that would produce besides neutralizing antibodies against neurotoxic amyloid-*β* oligomers, anti-inflammatory cytokines; preventing inflammation that aggravates AD. Because of age-linked immune decline, vaccines would be significantly more effective in preventing, rather than treating AD. Considering the amyloid-*β*'s role in tau's pathological hyperphosphorylation and their synergism in AD, the development of preventive vaccines against both amyloid-*β* and tau should be considered. Due to convenience and cost, vaccines may be the only option available to many countries to forestall the impending AD epidemic.

## 1. Introduction

Among the methods considered for treating Alzheimer's disease (AD), active immunotherapy (also known as vaccination) against amyloid *β* (A*β*) has been one of the most frequently tried [1]. This approach to AD therapy, which was first tested over 20 years ago with the AN1792 vaccine, has so far failed to show beneficial effects in humans. Indeed, the A*β* vaccines developed and tested to date induced production of antibodies that solubilize A*β* immobilized as plaque, but without tangible benefits on the patient's cognitive function. A consequence of the vaccine approach has been passive immunotherapy with monoclonal antibodies (mAbs) against A*β*. An approach based on the perception that administering targeted antibodies is more effective than trying to induce their production* in vivo*. After repeated setbacks with this approach, a new mAb, BAN2401, in the opinion of several researchers has shown to slow down the progression of early AD, creating cautious optimism in the field [2–5]. Another mAb, aducanumab, a replica of a protective antibody isolated from cognitively normal elderly humans, was found in a near-term phase 2 study to slow the progression of AD [3, 5, 6]. Yet, recently a long-term phase 3 clinical aducanumab study was ended, because of the unlikelihood that it would meet primary endpoints (http://investors.biogen.com/news-releases/news-release-details/biogen-and-eisai-discontinue-phase-3-engage-and-emerge-trials). The differences between the results of aducanumab's near-term phase 2 and long-term phase 3 clinical studies highlight the difficulties of treating AD after its onset, as discussed later in this review. Nonetheless, BAN24101 and aducanumab, differ from previous mAbs targeting monomeric A*β* [7], in that they recognize soluble cytotoxic A*β* protofibrils and oligomers (A*β*Os) [2, 3, 5–7], blocking their cytotoxicity. Their initial effects agree with reports from Klein et al. [8, 9] that the A*β*Os, and not the A*β* monomers or plaque, are the true culprits responsible for killing neurons. Subsequently, Klein et al. showed that antibodies against the A*β*Os rather than monomers, were the protective antibodies [10]. Later, Liu et al. [11] proved that nonprotective mAbs against regions like the A*β* N-terminal sequence (A*β*_1-15_) caused the release of cytotoxic A*β*Os that had been immobilized as plaque, an event known as the “dust-raising effect” [11, 12]. While anti-A*β* antibodies can solubilize plaque [13], this solubilization also can be achieved by methods like sonication [14]. Hence, it is apparent that plaque is a protection mechanism that immobilizes toxic soluble A*β*Os and A*β* protofibrils [15, 16]. Indeed, some people with high levels of A*β* plaque deposition are cognitively normal [17] further casting doubt on the putative role of plaque as the cause of AD.

From reports that antibodies against soluble A*β*Os, but not monomeric A*β*, protect neural cells against the cytotoxic effects of these amyloid-*β* aggregates, it is feasible to theorize that antibodies capable of neutralizing A*β*O cytotoxicity may be beneficial in preventing and/or treating AD. This objective apparently was achieved in transgenic mouse models with antibodies induced by vaccination, as well as the administration of certain mAbs directed against monomeric A*β*; results that could not be reproduced in humans. Therefore, it is likely that a vaccine capable of stimulating the production of antibodies blocking the cytotoxic properties of soluble A*β* aggregates could delay or prevent the onset of AD. Evidently, the reason for the reported clinical failure of AD vaccines is that they were designed to induce plaque removal rather than neutralize the cytotoxicity of A*β* soluble aggregates [12]. Besides, those vaccines had the wrong A*β*-derived antigen, i.e., A*β*_1-14_, combined with adjuvants that elicited an undesirable proinflammatory immunity rather than the required anti-inflammatory one [12]. The fact that soluble toxic A*β* aggregates have a large structural diversity that cannot be represented by the single linear epitope A*β*_1-14_ may explain why these vaccines failed to induce a protective immunity. A situation exacerbated by the fact that these vaccines were tested as therapeutic agents in elderly patients suffering of AD, where the immune system is weakened and the neurological damage may be beyond repair.

In this review, multiple issues concerning the development of effective AD vaccines will be discussed, taking into account past experiences with AD vaccines as well as new developments in AD passive immunotherapy. A significant body of information suggests that an effective AD preventive vaccine may be feasible, which would be radically different from the older vaccines. Considering the problems associated with aging, such as increase of inflammatory immunity and immunosenescence, it is doubtful that an effective therapeutic AD vaccine may be developed. Consequently, the AD vaccine development efforts should be focused on prevention rather than treatment.

## 2. Amyloid *β*, a Likely Vaccine Target

Since Alois Alzheimer reported a link between A*β* plaques and dementia, plaque deposits have become a hallmark of AD, considered by many to be the main causative agent of AD [16]. But recent information indicates that plaque may be a protection mechanism rather than the cause of AD [15–17]. Indeed, new evidence points to soluble A*β*Os and protofibrils, collectively A*β* aggregates, as the true AD etiological agents. Support for the role of A*β* aggregates as AD causative agents is provided by the work of Busche and Konnerth [18], which showed that AD's major functional impairments, e.g., excess neuronal activity and altered brain oscillations, are caused by A*β*Os in the absence of plaque. They also showed in transgenic mouse models that while mAbs against the A*β*_1-15_ region reduce plaque, they also aggravate neural disfunction [19]. This is likely a result of the released soluble cytotoxic A*β* aggregates from plaque, as explained in previous reports [11]. Bai et al. [20] provided further support for the proposition that A*β*Os and protofibrils, but not plaque, are AD's etiological agents. These scientists reported that abnormal dendritic calcium activity and synaptic depotentiation, which are associated with AD, occur before any A*β* plaque formation and that those abnormalities can also be induced by exogenous A*β*Os. The important role of A*β*Os in AD is further supported by the observation that A*β*Os isolated from the brains of AD patients caused impaired synaptic plasticity and memory when administered/injected in rats [21]. That coadministration of anti-A*β*O antibodies prevented those damaging changes, supports the use of immunotherapy as an option to prevent/treat AD. Accordingly, it is apparent that removal or solubilization of plaque by antibodies has no therapeutic value unless the cytotoxicity of soluble A*β*Os and A*β* protofibrils is neutralized, as appears to be the case with BAN2401 and aducanumab initial near-term results [3, 5, 6]. Since binding of antibodies to plaque results in plaque solubilization, the observation that BAN2401 and aducanumab reduce plaque was expected [6, 22].

Hence, the preliminary results obtained during the near-term phase 2 studies with aducanumab and BAN2401 point to new directions concerning the immunogen(s) needed to develop effective AD vaccines. Numerous reports have indicated that the immunogens needed to induce a protective immunity are the soluble A*β*Os and protofibrils, rather than either monomeric A*β* or plaque [8, 9, 23, 24]. These studies suggest that the critical epitope(s) is a conformational or three-dimensional one, rather than a linear one, such as the linear epitopes used in most AD vaccines tested to date except for AN1792 (Table 1). Support for an A*β* conformational epitope(s) is derived from studies using different soluble A*β* aggregates and the murine mAb precursor of the humanized mAb BAN2401. Indeed, this mAb recognizes the same common protofibril conformational epitope in all of the A*β* aggregates, regardless of differences in their amino acid sequences and N-terminal truncation [25]. Another important finding from the clinical and immunological studies with intravenous immunoglobulin preparations (IVIG) is that while natural antibodies (nAbs) recognize A*β*Os, they seldom recognize the A*β* monomer or its N-terminal region [23]. In contrast, vaccination with aggregated A*β*_1-42_ induces antibodies against both the oligomers and A*β*_1-15_ region [26]. Hence, A*β*O immunogens have been constructed with the A*β*_1-15_ region deleted, to prevent adverse antibody responses [27]; paradoxically, these undesirable immunoresponses are the ones being induced by practically all of the AD vaccines clinically tested or under development (Table 1).

Of significance for AD vaccines is that the immunoresponse elicited must be a systemic anti-inflammatory Th2 immunity, regardless of the presence of T-cell epitopes in the A*β* immunogen. Actually, those T-cell epitopes comprise the peptide sequences required to assemble the A*β*Os that stimulate the immune system to produce antibodies against the soluble A*β* aggregates. Indeed, nAbs and mAbs are being used in HIV-1 research to identify protective epitopes and create immunogens to induce a protective immunoresponse in HIV-1 vaccines [28, 29]. This situation supports the use of nAbs and mAbs, like aducanumab and BAN24021, to validate the selection of the soluble A*β* aggregates as immunogens for an AD vaccine. However, different from HIV-1 where there are no protective nAbs and the structures of the immunogens are still being determined, in AD the presence of protective nAbs and nature of the critical immunogens, i.e., A*β*Os and protofibrils, are known [9]. Hence, as with most vaccines, an effective AD vaccine should elicit a protective immunity rather similar to the natural one, but very unlikely a therapeutic one.

A consequence of the disappointing clinical results observed with A*β*-immunogens has been the development of alternative constructs that aim to mimic the epitopes responsible for A*β* neurotoxicity. Hence, besides the clinically unsuccessful A*β*_1-15_ derived immunogens [30, 31], certain constructs based on this sequence have been developed, which show beneficial effects in transgenic mouse models [32, 33]. Nonetheless, the fact that all of the clinically failed human vaccines and mAbs were found initially to be effective in AD transgenic mouse models raises serious concerns. The discrepancies between preclinical (mouse) and clinical (human) results may be explained by the fact that mice are more resilient than humans to the side effects of proinflammatory adjuvants, e.g., QS-21 and CpG [34], and that transgenic animals are artificial partial models of AD. Nonetheless, a new approach to develop novel immunogens is the use of A*β* oligomer-specific mimotopes, such as a dodecapeptide unrelated to A*β* and identified by Wang et al. [35]. This mimotope, as expressed on the surface of* Saccharomyces cerevisiae*, stimulates in mice the production of antibodies that recognize A*β*Os. However, if this dodecapeptide is expressed as a virus-like-particle or is conjugated to a protein carrier like KLH, it does not induce antibodies against A*β*Os [35]. These results indicate that both peptide and yeast are contributing to form a structure that mimics the A*β*O toxic conformational epitope. Though this new mimotope has beneficial effects in transgenic mice, it would need to be purified and its tridimensional-structure stabilized and tested in humans in order to become clinically acceptable. Nonetheless, that the protective antibodies induced by this mimotope target the A*β*Os rather than the monomeric forms strengthens the idea that soluble A*β* aggregates are one of the critical etiological agents in AD. The fact that the dodecapeptide forming the mimotope, when used by itself, can bind to A*β* monomers and inhibit the formation of cytotoxic A*β*Os [36], highlights its therapeutic potential to prevent the damaging A*β* aggregation leading to neurotoxicity.

A problem that has contributed to the uncertainties of AD research is A*β*'s pleomorphism and that its different assemblies correlate with distinct AD phenotypes [37, 38], a situation seldom encountered with other self-antigens. Apparently, the size and conformation of A*β*Os play a role in their cytotoxicity and, probably, though not being well understood, in inflammation [39]. This condition raises concerns about the therapeutic value of a limited number of mAbs, considering the potential heterogeneity of the neurotoxic A*β* conformers. Another topic that has raised additional doubts about the role of A*β* in AD has been the failure of *β* secretase 1 (BACE1) inhibitors to improve cognitive function in AD patients [40]. But BACE1 inhibitors, while reducing the production of A*β* and thus plaque formation, do not prevent the A*β* oligomerization leading to the production of the soluble cytotoxic A*β*Os, which occurs before plaque formation [18–20]. Although the BACE1 inhibitors have been tested in AD patients as a therapeutic, the current consensus is that their administration should start in a preventive mode during the presymptomatic phase of AD [40, 41]. Hence, targeting plaque formation while ignoring the early production of A*β*Os most likely would not result in beneficial effects. Indeed, a significant body of evidence [3, 8–10, 18–20, 24, 39] indicates that cytotoxic soluble A*β* aggregates are the relevant immunogen for a preventive AD vaccine, rather than the nontoxic monomers or plaque.

## 3. The Case for a Natural Protective Immunity

The existence of a natural protective immunity is essential for the successful development of an AD vaccine. It follows that the onset of AD could be prevented and/or delayed by eliciting or boosting that immunity. During the past two decades, a significant body of evidence has been gathered that supports the presence of such immune protection against AD in healthy individuals.

### 3.1. Substantiation of a Protective Immunity

The existence of protective autoantibodies against AD has been previously demonstrated preclinically and clinically using IVIG [42]. However, fractionation of IVIG by affinity chromatography yielded a fraction containing anti-A*β*O antibodies that when administered to AD transgenic mice improved their cognitive function to a higher degree than whole IVIG [23, 43, 44]. These anti-A*β*O antibodies bind to A*β* oligomers, but not monomers, and, different from antibodies induced by previous AD vaccines, they do not readily clear plaque [43]. The beneficial effects of the anti-A*β*O antibody fraction on cognitive functions in mouse models backs the presence of protective anti-AD natural antibodies (nAbs) that, due to immunosenescence, decrease with age when AD starts to appear [45]. Some neutralizing antibodies from IVIG and mAbs, like aducanumab and BAN2401, also facilitate plaque removal, besides binding to A*β* oligomers and blocking their cytotoxicity. The fact that plaque removal occurs with both protective and nonprotective antibodies [11, 19] shows that plaque clearing and protection against cytotoxic A*β*Os are unrelated phenomena. Plaque solubilization may be explained by the formation of A*β*-IgG complexes, where the more hydrophilic IgG disrupts the ionic-hydrophobic interactions holding the plaque together, regardless of where the antibody binds to A*β*. Incidentally, the fact that the protective anti-AD effects of whole IVIG preparations depend largely on the diversity of the IgG pools and their content of anti-A*β*Os neutralizing nAbs can explain the variability in the reported IVIG clinical results [46].

While the IVIG studies support the existence of a natural protective anti-AD immunity and its beneficial effects in delaying this disease's progression, the challenges of producing the IVIG needed to treat a large population would be burdensome [39]. An alternative method would be the use of chromatographically isolated nAbs with anti-A*β*O activity, instead of whole IVIG [23, 43, 44]; indeed, preclinical studies have shown the superiority of this approach compared to whole IVIG [43]. Yet, the logistic problems associated with the procurement of IVIG from which nAbs would be isolated and their administration to large populations will persist, a situation that backs the development of a preventive AD vaccine. Liu et al. [47] have described some of the characteristics needed for effective AD therapeutic antibodies, which can also be applied to the nAbs induced by preventive vaccination.

### 3.2. Inferences from the mAbs Clinical Studies

The encouraging preliminary results for BAN2401, as well as aducanumab [3, 5, 6], disagree with those from previous mAbs and AD vaccines which targeted the wrong A*β*-species, i.e., monomers and plaque, rather than the cytotoxic A*β* aggregates [12, 48]. Preliminary results from the BAN24101 study showed that at 18 months there was a dose-dependent slowing in cognitive decline from baseline [5]. At the highest dose, 10 mg/kg biweekly, the reduction was 30% on the ADCOM scale and 47% in the clinical decline, according to the ADAS-Cog scale [5, 49]. A concern with the BAN2401 study is the uneven distribution of APOE4 carriers, since the carriers were removed from the high-dose group, but not from the placebo one. But, in the opinion of some experts while that imbalance may create a possible bias, it does not seem to be a major concern [49]. Another issue with those results is that they may be artifacts due to the Bayesian statistical analysis used to interpret the clinical data rather than a frequentist statistical analysis [2]. But, a comparison of both methods shows the Bayesian analysis, which applies probabilities to statistical problems, to be more rigorous. By incorporating past information into the analysis, the Bayesian approach benefits from previous data [50], allowing deciding, based on early analysis of the responses, if a study offers strong signals of success or failure which may warrant its continuation or termination, respectively [51]. Different from frequentist statistics where a single parameter determines the clinical endpoint, the end point in the Bayesian adaptive phase 2 evaluation of BAN2401 is decided by the AD Composite Score (ADCOMS) [52]. ADCOMS, composed of 12 items that measure mild cognitive decline and none or limited functional impairment, apparently provides an improved sensitivity to determine minor cognitive changes [52], a reasonable approach considering the variety of pathological effects mediated by the cytotoxic A*β* aggregates. Hence, from the available information, it is possible to assume that the BAN2401 results are not a result of artifacts caused by the Bayesian statistics.

The fact that BAN2401 and aducanumab [53], like the nAbs isolated from IVIG, recognize A*β* soluble cytotoxic aggregates, apparently protecting neural cells from their damaging effects bolsters the role of immunity as a defense against AD. But for an effective protection, the antibody response must be targeted to the soluble aggregates, while avoiding the A*β*_1-15_ region, which causes the release from plaque of sequestered cytotoxic aggregates.

### 3.3. A*β* Clearance: Immunological Implications

An important issue in AD is the fate of the A*β*Os and IgG-A*β*O complexes, like those formed during plaque removal which occurs by several mechanisms. Soluble A*β* aggregates can be removed from the brain by enzymatic degradation and cellular uptake, transport across the blood–brain barrier (BBB) and blood–cerebrospinal fluid barrier (BCSFB), interstitial fluid (ISF) bulk flow, and cerebrospinal fluid (CSF) absorption into the circulatory and lymphatic systems [54, 55], processes that may take place concurrently. While BAN2401 and aducanumab appear to facilitate clearance of IgG-A*β*O complexes via Fc-dependent microglial phagocytosis [22, 56], extracellular A*β* also can be cleared by the other mechanisms [54]. The ‘peripheral sink hypothesis' is a safe antibody-mediated mechanism for reduction of A*β*O where anti-A*β* antibodies circulating in the blood bind and sequester plasma A*β*, thus preventing its entry into the brain [47]. By disrupting the equilibrium across the BBB, the A*β* content inside the brain would remain low, preventing the formation of toxic A*β*Os. However, the peripheral sink clearance is not supported by results obtained with BACE1 inhibitors, where lowering A*β* production did not improve cognitive function, possibly because these drugs were used as a treatment, rather than in a preventive mode before the onset of disease [40, 41].

The need for Th2 immunity in AD immune therapy is emphasized by the association of the microglia phagocytic activity with the M2 activation phenotype, which is anti-inflammatory, and that such activity can be decreased by proinflammatory cytokines [57]. Although binding of IgG's Fc region to the high-affinity human Fc-receptor on microglia, Fc*γ*R1, could promote IgG-mediated inflammation [55], that appears not to be the case with BAN2401 or aducanumab, as shown by safety studies [2, 6]. Thus, it is feasible that the Fc regions of these mAbs bind to low-affinity IgG Fc*γ*Rs, which are less likely to induce an inflammatory immunoresponse [58]. Important for AD vaccine development is that A*β*Os and perhaps protofibrils can initiate A*β*O neurotoxicity by interacting with the Fc*γ*RIIb receptor on neurons, a damaging effect that may be inhibited by antibodies blocking A*β*O interactions with that receptor [59]. The effects of interactions between IgG's Fc and Fc*γ*R on the type of immunity, i.e., Th1/Th17 or Th2, bolster the prerequisite that anti-AD vaccines must induce a systemic anti-inflammatory Th2 immunity, an immunity that would allow the production of neutralizing antibodies and clearance of A*β* by microglia's phagocytosis, without adverse Th1 proinflammatory immunoresponses.

The safety of nAbs against cytotoxic A*β*Os is supported by the fact that clinical studies have failed to show differences in the rate of serious adverse effects among individuals receiving IVIG and 0.9% isotonic sodium chloride solution [60]. Based on the information available from clinical studies with IVIG-derived nAbs, and mAbs like aducanumab and BAN-2401, it is evident that safe and effective AD preventive vaccines should induce a sole anti-inflammatory Th2 immunity. However, some vaccines under development use proinflammatory adjuvants to elicit an immunoresponse, a strategy that ignores the damaging effects of an inflammatory immunity on AD progression [61, 62].

## 4. AD Vaccines: Clinical Outcome

**Figure 1:**
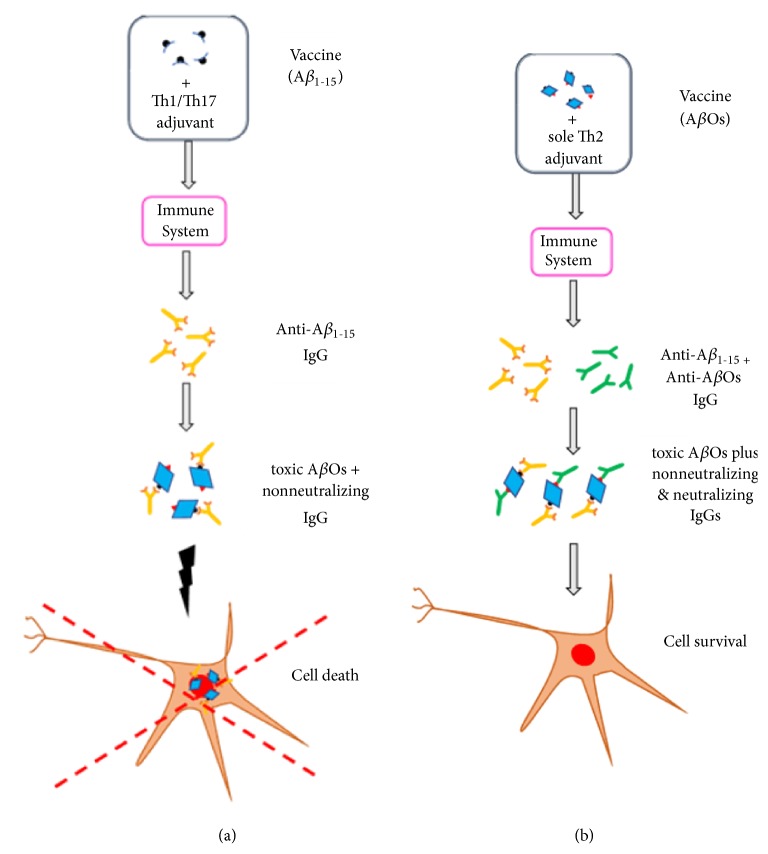
*Immune response induced by different Aβ vaccines*. (a) Vaccines having the A*β* N-terminal region (A*β*_1-15_) as an immunogen induced the production of antibodies (orange) that while binding to A*β* monomers and A*β*Os, are not protective. Similar to the ineffective mAbs that bound A*β*_1-15_, these antibodies cannot neutralize the cytotoxic A*β*Os, and protect neural cells from death. That these antibodies release toxic A*β*Os, which were immobilized as plaque, increase their harmful levels in the brain. Use of proinflammatory adjuvants in these vaccines, induces a systemic Th1/Th17 immunity, which increases the damage associated with AD. (b) Vaccines having A*β*Os as an immunogen and a sole Th2 adjuvant, elicit an anti-inflammatory immunity characterized by the production of antibodies against the A*β* N-terminal region (black) and A*β*Os cytotoxic conformational epitopes (red). Different from previous AD vaccines, which only induced production of anti-A*β*_1-15_ antibodies (orange), the antibodies against the A*β*Os' cytotoxic epitopes will be neutralizing ones (green), thus protecting the neural cells from death. Since the elicited neutralizing antibodies are against different conformational epitopes, it is feasible to expect some cooperative effects among the different antibodies. A synergism that would increase their protective effects on neural cells. Stimulation of a sole systemic anti-inflammatory immunity will prevent and/or delay some of the AD pathological changes associated with inflammation.

The apparent near-term benefits of the mAbs aducanumab and BAN2401 on prodromal AD as shown by the phase 2 clinical studies [3] implies that the cytotoxic soluble A*β* aggregates are the etiological agents of AD, and also explains the disappointing results of past AD vaccines [63]. Due to their immunogens derived from the A*β*_1-15_ region, the B-cell epitope, those vaccines induced antibodies against the A*β* monomers and exposed N-terminal region in cytotoxic A*β*Os and protofibrils (Figure 1(a)). Although such antibodies recognized A*β* aggregates, they failed to neutralize the conformational epitope(s) responsible for toxicity. This resulted in plaque removal by solubilizing the immobilized cytotoxic A*β*Os [11, 19], but without neutralizing them [48]. A special situation was observed with the AN1792 vaccine, which had aggregated A*β*_42_ plus the strongly proinflammatory adjuvant QS-21. This vaccine caused meningoencephalitis during phase 2 clinical studies, which prompted the termination of the study [64]. Evidently, the damaging proinflammatory Th1/Th17 autoimmune response induced by QS-21 [65] was boosted significantly higher during phase 2 by the addition of the detergent polysorbate 80 to the vaccine formulation [12, 48]. One outcome of this study has been the belief that despite its problems, AN1792 reduced plaque and also improved cognitive decline as compared to the group receiving placebo with QS-21 [66]. But, as reported by Von Bernhardi [67], those receiving placebo with QS-21 had an abnormally high rate of cognitive decline, i.e., 6 to 7 points loss in the Mini Mental Status Examination (MMSE), which is greater than the average loss of 3.5 to 4 points for AD patients. These results show that a systemic proinflammatory immunity, like that induced by QS-21, exacerbates AD even in the absence of an autoimmune response [68].

Clinical studies have shown that vaccines having A*β*-derived immunogens without T-cell epitopes, but formulated with Th1 adjuvants, despite exhibiting beneficial effects in AD transgenic mouse models; they did not benefit humans [30, 31]. Another factor contributing to these vaccines' poor performance and side effects s has been the absence of effective sole anti-inflammatory Th2 adjuvants [34, 69]. Alum, the traditionally assumed sole Th2 adjuvant induces production of proinflammatory cytokines, activates complement, and stimulates the activation of monocytes [70, 71], all adverse proinflammatory activities in vaccines against proteopathies. The presumed solution to the damaging T-cell mediated autoimmunity from past AD vaccines, had been to use the B-cell epitope A*β*_1-15_ or peptides from that sequence, conjugated to carrier proteins or peptides [72, 73], combined with Th1 adjuvants to yield high antibody titers (Table 1). This solution ignores that these adjuvants induce a systemic proinflammatory immunity, regardless of the absence or presence of an immunogen, as shown by the detrimental effects of QS-21 alone on the cognitive function of individuals affected by AD [67]. Hence, it is premature to conclude from the past failures of AD passive and active immunotherapy that A*β* is the wrong therapeutic target for vaccine development. Actually, the poor results are those expected for AD vaccines having the incorrect immunogen and adjuvant, as discussed in this review.

A problem affecting AD vaccine development has been the lack of information about their composition. For instance, the immunogen for most AD vaccines had been short A*β* peptide sequences linked to a carrier. But crosslinkers may have groups like maleimide, which due to their high immunogenicity, suppress the immune response against the peptide [48, 74]. The fact that vaccines like Affitope AD02, and presumably CAD106, have peptides mimicking A*β*_1-15_, conjugated to a KLH or virus-like-particle carrier by maleimide crosslinkers, indicates that the potential maleimide's interference with the immunoresponse was not considered [48, 74]. Thus, there is little information to explain these vaccines' failures, a deficiency that may have contributed to the disappointments, since all the vaccines had related immunogens based on the A*β*_1-15_ region (Table 1) [31, 72, 75]. The results for the vaccines ACC-001 and CAD106 [76, 77], having immunogens derived from A*β*_1-15_, show that they induced antibodies against that sequence which removed plaque, causing brain volume's loss. But apparently those antibodies did not neutralize cytotoxic A*β*Os, which cause the death of neural cells [11, 19, 20]. Thus, the released cytotoxic A*β*Os may aggravate the course of AD in the long-term (Figure 1(a)). An unexpected result of the ACC-001 vaccine study is the absence of cognitive differences among individuals receiving saline solution, QS-21-only, or the immunogen plus QS-21 [76]. These results contrast those from the AN1792 study, where QS-21-alone worsened cognitive functions as compared to the decrease in untreated AD patients [67]. This discrepancy may be explained by the fact that both studies used the same 50 *μ*g dose of QS-21 as a placebo, but with and without polysorbate. It is well-known that addition of some detergents increases QS-21 adjuvanticity by several folds, inducing a higher and damaging systemic proinflammatory Th1/Th17 immunity [12, 48].

A vaccine undergoing clinical studies, UB-311, has an immunogen comprising the B-cell epitope, A*β*_1-14_, linked to two different T helper (Th) peptides [33, 78]. It has been reported that UB-311, which contains alum plus CpG ODN as an adjuvant, induces Th2 immunity [78]. But, the selection of CpG as an adjuvant is unexpected, since the CpG motif is a TLR9 ligand well-known to induce a strong proinflammatory Th1 immunity against cancer and pathogens [79, 80]; an immune response that would antagonize the desirable effects of an AD vaccine. Actually, another vaccine containing A*β*_1-14_ linked to pathogen-derived Th epitopes as an immunogen and the adjuvant *δ*-inulin/CpG ODN, induced both strong Th1 and Th2 immune responses, rather than a noninflammatory Th2 immunity [81]. Hence, the reported Th2 immunoresponse induced by UB-311 might be due to the Th peptides to which the A*β*_1-14_ is linked [78]. The antibodies induced by UB-311 in humans are reported to bind A*β* monomers, oligomers and fibrils, inhibiting fibril formation and associated cytotoxic effects [78]. According to the investigators, the results of the cognitive studies, while being apparently promising, are not statistically significant due to the small number of patients.

Consequently, it is evident from the poor results of AD vaccine clinical studies that progress in this area has been negligible, most likely the result of the vaccine formulations used [12, 34, 48]. The majority of AD vaccines to date have used the A*β*_1-15_ region as the immunogen. This selection ignores that the anti-A*β*O nAbs shown to protect neural cells from the toxic effects of A*β*Os and fibrils, and improve cognitive functions in AD transgenic mice, recognize the A*β* mid-/C-terminal rather than the A*β*_1-15_ region. [23, 44]. Unlike most self-antigens in autoimmune diseases, A*β* is pleomorphic and its different assemblies correlate with distinct AD phenotypes that may cause various pathological effects [37, 38]. Thus, vaccines targeting only the A*β*_1-15_ region may be of no value, since effective AD vaccines should target other relevant epitopes found in A*β* oligomers, an approach that requires using the whole A*β* as an immunogen, including the T-cell epitopes' sequences, to form the different conformational epitopes of A*β*Os and fibrils (Figure 1(b)). This comment may be also applied to mAb therapy, where the antibody recognizes a single conformational epitope. Finally, effective AD vaccines will require sole anti-inflammatory Th2 adjuvants that inhibit detrimental, inflammatory Th1/Th17 immune responses, while inducing the DCs responsible for regulating immunity, towards a tolerogenic phenotype, thereby biasing the immune response towards an antibody one [82, 83].

## 5. Feasibility of an Effective AD Vaccine

An examination of the relationships between A*β* and AD reveals a complexity beyond that of the conventional theory that an excess of A*β* and plaque deposits are AD's causative agents [7, 9, 24], a theory clouded by the fact that all of the studies attempting to treat AD by plaque removal and/or lowering A*β* levels in the brain have failed to show beneficial effects clinically. Hence, some authors have raised questions about the validity of the A*β* hypothesis, as well as the design of the studies, e.g., the drugs being tested and target population [84], which may have contributed to the poor results. Yet, it is evident that those studies did not take into account the damaging effects that soluble A*β* aggregates, i.e., A*β*Os and protofibrils, inflict on neural cells leading to AD [18–20, 37, 39]. Thus, based on the information derived from the IVIG's anti-AD nAbs, the initial near-term results with the mAbs aducanumab and BAN2401, and the structural characteristics of the different A*β* aggregates, the likelihood of developing a different but useful preventive AD vaccine seems reasonable. Here, the characteristics of the key components of such a vaccine, i.e., the immunogen and the adjuvant, will be addressed.

### 5.1. Amyloid *β* Derived Immunogens

While AD vaccine development has been biased towards the use of the A*β*_1-15_ (B-cell epitope) as a safe immunogen (Table 1), a large body of evidence points to the A*β* soluble aggregates as the proper antigen. Actually, immunogens based on the A*β*_1-15_ peptide would induce a damaging antibody response [11, 47] (Figure 1(a)). Instead, a suitable immunogen might contain a wide distribution of A*β*Os, from ~ 10 kDa to more than 100 kDa, i.e., the cytotoxic ones [27, 85–87]. But, since oligomerization depends on both concentration and time, a long-term incubation may alter the vaccine formulations with concomitant changes in their immune stimulatory properties. One solution to the instability problem could be cross-linking of the oligomeric forms in a manner that does not alter their immunogenic properties. Glutaraldehyde [27] has been used to stabilize these oligomers, but while sound, this approach may have complications due to glutaraldehyde polymerization. An alternative method is the use of 1,5-difluoro-2,4 dinitrobenzene (DFDNB), a cross-linking agent that reacts with lysine's amino groups [88]. In fact, DFDNB cross-linked A*β*Os are particularly potent in inducing memory dysfunction in mice, an AD associated disorder.

**Figure 2:**
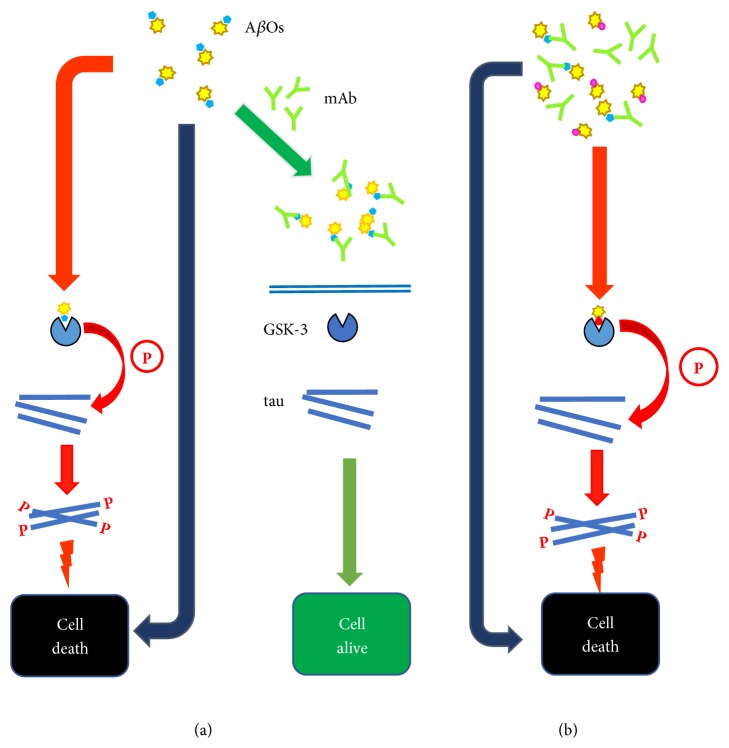
*Effects of mAbs targeting AβOs on their neurotoxicity*. (a) Neurocytotoxic A*β*Os cause damage by acting at the neural cell membrane level and upon uptake, intracellularly (red arrows). But A*β*Os also amplify their cytotoxicity by activating tau kinases, e.g., GSK-3, which hyperphosphorylate tau (blue rods). Like A*β*, the aberrant phosphorylated tau (P) forms oligomers that are cytotoxic; but, the mechanism(s) by which tau-P causes neurodegeneration is still unclear. The A*β*Os' damaging effects may be prevented by mAbs (green), which upon binding to A*β*Os block the conformational epitopes responsible for cytotoxicity and activation of tau kinases. This situation would explain the near-term benefits achieved by using mAbs like aducanumab. (b) The presence of A*β*Os as various conformers having different epitopes explains why the protection provided by mAbs against A*β*Os is limited to near-term. While some cytotoxic A*β*Os are being neutralized by the administered mAbs (green), new ones showing diverse epitopes (magenta) and thus able to escape neutralization by those mAbs are being produced. However, these new A*β*Os are cytotoxic and hence able to damage neural cells (red arrows), as the previously neutralized A*β*Os. Consequently, the development of these new populations of cytotoxic A*β*O conformers (magenta) would explain why the protective effects of mAbs, e.g., aducanumab, are limited in duration.

Since the polymorphic A*β*Os may show a diversity of pathological effects, it would be advantageous if the immunogen presents to the immune system an array of A*β* conformers responsible for those effects, to induce a broad antibody response. Indeed, it has been suggested that similar to vaccines against pathogens, the various A*β* conformational epitopes are comparable to strains showing different neurotoxicity [38, 89]. The presence of different conformational epitopes would explain the polymorphism and various pathophysiological effects of A*β*Os of similar size [85]. From the available information, it is evident that small A*β*Os, e.g., dimers, to tetramers, are extremely cytotoxic and serve as building blocks for larger oligomers [85]. The complex composition of A*β* based immunogens is confirmed by reports of cell membrane disruption by A*β*Os and the structural characteristics of oligomers with increased affinity for cell membranes [90]. Hence, it is obvious that immunogens derived from the A*β*_1-15_ region cannot produce the conformers needed to induce a protective anti-A*β* antibody response capable of neutralizing the various cytotoxic A*β*Os (Figure 1(b)). This conclusion must also apply to passive immunotherapy, where mAbs recognize a single epitope (Figure 2). This limitation may be minimized by using a combination of mAbs against diverse epitopes, a costly solution.

Therefore, effective immunogens based on A*β*Os should have a composition that includes various oligomeric forms presenting different conformers. Although the methods to prepare those oligomers, i.e., recombinant DNA technology and organic synthesis, are available, the challenge would be to produce them in a reproducible manner to elicit a useful immune response against a set of dominant A*β*Os, a difficult task as proven by the fact that the polymorphism of different A*β*O preparations largely depends on the making and purification of the A*β*_42_ polypeptide [91]. Because of the effects of excipients on the immunogen and adjuvant properties, utmost care must be taken in their selection to avert injurious situations like those observed with the AN1792 vaccine [12, 48]. Finally, characterization of the immunogen must employ methods like electron microscopy, NMR, and X-ray crystallography to assess the conformation of the multiple A*β*Os [92].

### 5.2. Adjuvant's Role on the Immune Response

Adjuvants by acting on DCs and T-cells modulate the immune response to a proinflammatory Th1/Th17 or anti-inflammatory Th2 immunity. An effective AD vaccine must elicit a sole anti-inflammatory Th2 immunity, to avert damaging inflammatory T-cell mediated autoimmune responses, like those induced by the AN1792 vaccine. This requirement resembles that for vaccines against T-cell mediated autoimmune diseases, which also need a sole anti-inflammatory immunity [93]. Therefore, an obstacle in AD vaccine development has been that the available adjuvants, e.g., QS-21, CpG DNA, monophosphoryl lipid A and dsRNA, are mainly TLR ligands that stimulate a proinflammatory Th1/Th17 immune response, irrespective of the presence of T-cell epitopes in the immunogen [30, 31, 34]. A fact that is seldom recognized in AD vaccine development is that the adjuvants used in the current AD vaccines are largely proinflammatory ones (Table 1). Hence, vaccination methods that may not need adjuvants are being investigated, like DNA vaccines, where the immunogen is administered as a DNA gene encoding for the A*β* antigen [94]. After a transient transfection, the body's cells express the A*β*, inducing a noninflammatory immune response against toxic A*β*, a response that also reduces tau pathology [94]. While promising, a problem might be the deteriorating effects of immunosenescence on the immune system; i.e., the DNA vaccine's efficacy against infectious agents decreases in aged mice [79]. This immune decline may be ameliorated by coadministration of the adjuvant CpG ODN, as shown in aged mice. But, CpG DNA preferentially promotes a Th1 differentiation, which is proinflammatory and damaging in the case of AD [95].

Another approach to AD vaccines is the* ex vivo* use of DCs sensitized by an A*β*_42_ having mutated T-cell epitopes. Unlike normal A*β*_42_, an A*β*_42_ having mutated T-cell epitopes can induce an antibody response without inflammation [96]. Because of their beneficial effects in AD transgenic mice and lack of proinflammatory side effects, the use of DCs as a vaccination method has been proposed to prevent or treat AD [96, 97]. This approach requires isolating the patient's DC precursor cells, which after* ex vivo* maturation and stimulation with the mutated A*β*, are reinjected back into the patient, a complex and costly method for use in large populations in a preventive mode. Yet, use of DCs seems to avert some of the problems associated with the classic AD vaccines, like inflammatory responses. Indeed, the DC's role in regulating the brain's inflammatory status and the advantages of DC-based vaccines to treat AD have been the subject of extensive discussion [98]. One conclusion is that DCs' function may be modulated by adjuvants, a strategy that may increase the efficacy of DC vaccines for AD therapeutic purposes.

Although alum has been considered a Th2 adjuvant and used as a safe anti-inflammatory adjuvant in some AD vaccines, it shares many properties with Th1 adjuvants [70]. Alum's mechanism of action is complex, involving Th1 and Th2 associated processes, inducing a state of so called proinflammatory preparedness [70, 71]. Actually, the type of immunity induced by alum, seems to depend on numerous factors [71]. This situation raises questions about its use in AD vaccines, alone or combined with other adjuvants [33, 34]. A valuable option is the use of sole Th2 adjuvants, like the fucosylated glycoside QT-0101 [34, 69], to boost the immunoresponse of DC-based vaccines and bias T-cell activation towards Th2 immunity [98]. In fact, any protein antigen attached to a fucosyl glycan, like LNFPIII, would bind the lectin receptor DC-SIGN on DCs [99], which acts as an endocytic receptor and deliver the immunogen for intracellular processing by DCs. Binding of a fucosyl residue to DC-SIGN would induce in DCs a tolerogenic phenotype, which biases the response towards Th2 immunity [82, 83]. Delivery of the immunogen to the same DC being activated by the fucosyl residue binding to DC-SIGN after repeated immunizations will favor the production of antibodies with enhanced neutralizing properties [100]. Also, use of adjuvant-immunogen complexes would allow the* in vivo*, rather than* ex vivo*, targeting of immunogens to maturing DCs, a process that would significantly improve vaccination and allow for its use at a large scale in a preventive mode.

The glycoside QT-0101 has some advantages over the other Th2 adjuvants. Because of its amphipathicity and detergent-like properties it should form complexes with protein antigens, which are stabilized by a combination of ionic/hydrophobic interactions. This characteristic eliminates the need for the covalent linking used with LNFPIII [99]. The formation of A*β*O/QT-0101 complexes could also help to preserve the structural integrity of the A*β*Os, by avoiding their further aggregation. In effect, similar to other saponin adjuvants, addition of a detergent, like polysorbate, to QT-0101 vaccine formulations, may alter its adjuvanticity [69]. Hence, the ability of QT-0101 to inhibit Th1/Th17 immunities while eliciting a systemic Th2 anti-inflammatory immunity [65, 69] would help to slow the neurodegenerative process that may be exacerbated by inflammatory signaling responses [101].

## 6. Outstanding Issues

Increasing evidence shows that plaque is a protection mechanism that immobilizes cytotoxic A*β*Os rather than a causative agent of AD. The fact that high brain plaque buildup occurs in normally cognitive aging people and that some AD related pathophysiological effects befall in the absence of plaque [15–17] questions the role of these structures in AD. Actually, the process of removing plaque by releasing damaging A*β*Os could aggravate the course of this disease. Thus, the assessment of plaque removal as an indicator of AD treatment efficacy should be reconsidered in view of the available information.

While the current evidence shows that soluble A*β* aggregates are the neurotoxic agents leading to AD, only two studies, aducanumab and BAN2401 [2, 4], have targeted these aggregates. With the exception of AN1792, AD vaccine development has been essentially focused on the A*β*_1-15_ region, despite the fact that antibodies against that region may aggravate AD [11]. Considering the convincing evidence indicating that A*β*Os, and not monomeric A*β*, induce the damaging effects linked to AD, it would be logical to reconsider the relevance of past vaccine studies in the development of preventive vaccines against AD.

Until recently, AD vaccine development has been largely based on the belief that immunogens lacking T-cell epitopes, e.g., antigens like A*β*_1-15_, can be used safely with adjuvants that induce a systemic proinflammatory immunoresponse. But this belief cannot be applied to A*β*Os since oligomer formation requires the entire A*β* amino acid sequence, including B and T-cell epitopes. Besides, a proinflammatory immune response will exacerbate the damaging effects associated with AD. Accordingly, a useful AD vaccine would require adjuvants that induce a sole anti-inflammatory Th2 immunity, while inhibiting without abrogating proinflammatory immunity, needed for protection against infectious agents and cancer. This constraint makes the identification of new sole Th2 adjuvants critical for the development of vaccines against AD and other proteopathies.

It is apparent that AD is a multifactorial syndrome that results from independent, age-related pathologies, which justifies the use of a multiprong therapy approach [84]. Thus, preventive immune therapies may include more than one target. A review of the potentially most important causative agents of AD shows that, besides A*β*, the tau protein is also critical. Indeed, the available information strongly suggests that immunotherapy is an effective option to deal with the effects of aberrant tau [89]. Hence, as it would be discussed here, the question is how to block the damaging effects of both the abnormal A*β* and tau forms to prevent and/or delay the onset of AD.

## 7. Discussion and Perspectives

The preliminary phase 2 encouraging near-term clinical results of AD immunotherapy with BAN2401 and aducanumab support the development of preventive vaccines against this disease. Vaccines that induce an anti-inflammatory Th2 immunity with production of neutralizing antibodies against the cytotoxic A*β* soluble aggregates to protect neural cells from death. An immunoresponse that none of the clinically tested vaccines were able to induce, despite the seemingly convincing results from transgenic mouse models [12]. Misleading results that led the development of vaccines and mAb immunotherapy to focus on the N-terminal region of the A*β* monomer rather than the most likely cause of AD, the A*β*Os. A condition aggravated by the vaccines' induction of a proinflammatory immunity, which would exacerbate the course of AD. Thus, effective AD vaccines will require adjuvants that induce a sole systemic Th2 anti-inflammatory immunity and immunogens that have the dominant cytotoxic conformers for presentation to the immune system [34].

Actually, the fucosylated glycoside QT-0101 has several advantages; its fucosyl residue can bind to DC-SIGN, inducing an intracellular signaling that biases the response to Th2 immunity, while inhibiting the proinflammatory Th1/Th17 immunities [65, 69]. A stable immunogen and QT-0101 complex would allow* in vivo* targeting of the immunogen to DC-SIGN and its uptake by receptor-mediated endocytosis, followed by its intracellular processing. This pathway would induce a systemic Th2 immunity with production of anti-inflammatory cytokines and antibodies that neutralize cytotoxic soluble A*β* aggregates. A response that would improve with repeated vaccinations, which favor the selection of higher-avidity antibodies with better neutralizing properties [102]. The immune response will be influenced by the adjuvant, particularly if it facilitates delivery of the antigen to DCs [100], as QT-0101 does. Like previous anti-A*β* antibodies, the newly induced antibodies would most likely remove plaque, regardless of their effects, underscoring the fact that plaque removal is a phenomenon unrelated to protection against AD.

Although AD vaccines have been considered as therapeutics, the age-associated immune decline and resulting disturbances in antibody production make that option unlikely. While patients' immune competence may be of no concern in mAb therapy, it is critical in vaccination, a situation complicated by the development of a damaging chronic inflammation associated with aging [103]. Thus, immunizations to boost natural immunity should start years before the immune decline that occurs by age 65 [104]. Most likely, earlier immunizations may be needed for those having known risk factors, like carrying the APOE4 gene and diagnosis of type 2 diabetes [105], which may develop AD before 65 years of age. A vaccine's effectiveness in overcoming immunosenescence will solely depend on the adjuvant component. Hence, effective adjuvants should ameliorate some of the changes caused by immunosenescence, like the loss of ligands and receptors on T-cells and DCs, and the capacity to deliver the costimulatory signals needed for T-cell activation [106]. While it is doubtful that vaccination would totally prevent the onset of AD, boosting the protective anti-AD immunity would most likely delay the onset of this disease by several years.

Although soluble A*β* aggregates play a critical role in AD development, the likely role of tau protein in this disease cannot be ignored. Current information shows a close but intricate relation between tau and A*β*, with potential synergy in causing neurodegeneration [107]. For instance, an abnormal phosphorylation of tau renders this protein neurotoxic [108], a modification induced by monomeric A*β*_1-42_ [109], which is more prone to oligomerization than the shorter A*β*_1-40_. Meta-analysis studies show that cognitive decline is more affected by the increase in phosphorylated tau than that of A*β* soluble oligomers [110]. It also validated that plaque or immobilized A*β* has no role in the cognitive decline, strengthening the opinion that plaque does not cause AD. Application of statistical methods indicates that A*β*Os have a strong influence in activating kinases for tau's phosphorylation [106, 110], most likely by increasing the activity of glycogen synthase kinase 3*β* (GSK-3) [111]. An enzymatic step that would result in an amplification of tau's phosphorylation process, an expected outcome because enzymes are biological catalysts. This process may explain the long-term disappointing aducanumab phase 3 clinical results; i.e., while blocking of A*β*Os initially reduces tau phosphorylation, it cannot stop it (Figure 2(a)). Besides, A*β*'s polymorphism [37, 38] may yield new conformers that cannot be blocked by aducanumab [37–39]; hence, they would be able to induce a damaging enzymatic tau phosphorylation (Figure 2(b)), a situation that may explain the lack of long-term AD protection in the presence of aducanumab, an observation that may apply to other mAbs.

That GSK-3 is under the control of soluble A*β*Os, reinforces the fact that their neutralization will halt the damaging tau's phosphorylation process (Figure 2(a)), an argument that supports the concept of a preventive vaccine to inactivate soluble A*β*Os, blocking a cascade of damaging events. Concerns regarding the use of phosphorylated tau as a therapeutic target stem from the fact that several tau phosphorylation sites are linked to its normal physiological role. To obviate this difficulty, Kovak et al. [112] identified an epitope unique to the aberrant tau forms which is phosphorylation independent. This epitope has been used to develop a vaccine that interferes with the tau-tau interactions essential for tau's pathological effects in AD. Considering the apparently close interactions among tau and A*β* and their potential synergism, an effective approach would be to block both of them, using mAb immunotherapy and/or vaccination, in order to prevent or delay the onset of AD.

It is estimated that by the year 2050 there will be worldwide 125 million AD cases, emphasizing the importance of developing an effective AD vaccine. Seventy-five percent of the world's cases are predicted to occur in developing countries from Asia and Latin America, while the incidence of AD in the developed countries is expected to stabilize or show a slight decrease [Prince MJ. World Alzheimer Report 2015: The Global Impact of Dementia. London: Alzheimer's Disease International; 2015. https://www.alz.co.uk/research/worldreport]. Epidemiological data shows that the onset of AD in Latin Americans occurs on the average seven years earlier than in white Americans and that they also have a higher incidence of diabetes, which is an AD risk factor [113, 114]. Yet, many countries lack the public health infrastructure and financial resources needed for prevention/treatment campaigns based on mAbs, which are considered costly even in wealthier countries. An approach to deal with this crisis would be to develop preventive vaccines against AD that would induce neutralizing antibodies against the cytotoxic A*β*Os, rather than removing plaque and increasing the pool of cytotoxic A*β* soluble forms. By delaying the onset of AD, an effective vaccine would mitigate the negative impact of this disease on the socioeconomic fabric of many countries.

## Figures and Tables

**Table 1:** Alzheimer's disease vaccines at the clinical or preclinical stage.

Vaccine	Immunogen	Carrier	Adjuvant	Immunity^◊^	Status

AN1792	A*β*_1-42_	None	QS-21	Th1/Th2	Terminated
ACC-001	A*β*_1-7_	CRM197*∗*	QS-21	Th1/Th2	Terminated
Affiris AD02	mimic A*β*_1-6_	KLH	Alum*∗∗*	Th2	Terminated
CAD106	A*β*_1-6_	VLP^§^	None	-* *-* *-* *-	Phase 2
V950	A*β* N-terminal	ISCOMATRIX	Quil A	Th1/Th2	Terminated
UB-311	A*β*_1-14_	UBITh^¶^	CpG+Alum	Th1/Th2	Phase 2
Advax^CpG^	A*β*_1-11_	Th epitopes	*δ*-inulin+CpG	Th1/Th2	Pre-clinical
ABvac40	C-term. A*β*_40_	KLH	Alum*∗∗*	Th2	Phase 2
ACI-24	A*β*_1-15_	Liposomes	MPLA^▲^	Th1/Th2	Phase 2
Lu AF20513	[A*β*_1-112_]_3_	Tetanus toxin	-* *-* *-* *-	-* *-* *-* *-	Phase 1
AADvac1	Tau-C- _294-305_	KLH	Alum*∗∗*	Th2	Phase 1

(◊) Immunity means the type of immunity induced by the adjuvant, as reported in the literature and not the one described for the vaccine in question. (*∗*) CRM197 nontoxic diphtheria toxin. (*∗∗*) Alum a traditionally assumed Th2 adjuvant has many proinflammatory properties [67, 68]. (§) VLP are virus-like-particles derived from Q*β* phage. (¶) UBITh is a proprietary set of T helper epitopes derived from MVT, PT and TT [75]. (**▲**) MPLA monophosphoryl lipid A.
